# Progress in Maternal, Newborn, and Child Health in Cities in Sub-Saharan Africa: Are Wide Inequities Holding Back Cities?

**DOI:** 10.1007/s11524-024-00936-x

**Published:** 2024-10-23

**Authors:** Cheikh Mbacké Faye, Blessing Mberu, Ties Boerma

**Affiliations:** 1 African Population and Health Research Center, West Africa Regional Office, Dakar, Senegal; 2 African Population and Health Research Center, Nairobi, Kenya; 3 University of Manitoba, Winnipeg, Canada

Over the last few decades, sub-Saharan Africa (SSA) has experienced unprecedented urbanization, largely driven by rural-to-urban migration in pursuit of better living standards and health outcomes. Urban areas, especially capital cities, have traditionally been viewed as centers of progress and development, drawing migrants seeking improved health and living conditions [1, 2]. This has significant implications for national progress toward achieving the Sustainable Development Goals (SDGs) for maternal, newborn, and child health (MNCH).

However, cities concentrate on risks and hazards to health. The migration surge to urban Africa has outpaced the capacity of governments to effectively plan and provide essential services, leading to the proliferation of urban informal settlements. With estimates suggesting that two-thirds of urban dwellers in SSA live in slums or slum-like conditions [3, 4], the health consequences for these populations are dire. Children living in slums are more likely to experience early childhood mortality, malnutrition, and higher rates of childhood illnesses compared to those in rural areas [5].

The situation presents complex challenges for MNCH. Despite the common perception of cities as centers of progress and development, there are alarming signs of deceleration in the coverage of essential health interventions and increases in child mortality rates, particularly in comparison to rural areas [6, 7]. In fact, the “city advantage” in health outcomes appears to be eroding [8]. Coverage of essential health services has stagnated, and child mortality rates in many urban settings, including many capital cities, are rising relative to rural populations. Analysis of select interventions across countries reveals that national progress in increasing coverage rates is primarily driven by rural improvements, while urban areas continue to lag behind [9].

National household surveys, such as DHS and MICS, are the principal data sources for monitoring progress in child survival, nutrition status, and MNCH intervention coverage across countries. These surveys often allow for assessments of urban areas, distinguishing between the main metropolis or capital city and other urban zones. However, further disaggregation to capture insights into the urban poor is less common, primarily due to sample size limitations. Additionally, identifying slum populations can be challenging due to the GPS scrambling of survey cluster locations to protect individual identities. This, combined with the limited focus on urban MNCH from both a monitoring and programmatic perspective, are major gaps that must be addressed in national data collection initiatives.

In this special issue, we tackled these urban health inequity issues through a collection of eleven papers that offer critical insights into the state of MNCH across the rapidly expanding urban landscapes of SSA. This effort is part of a collaboration between the Countdown to 2030 initiative and eleven SSA countries, aiming to generate evidence to inform policy and interventions that address health inequities within urban settings. By focusing on MNCH, the special issue contributes to broader goals of achieving universal health coverage and advancing the SDGs.

This special issue highlights the largest cities in SSA, many of which are capital cities. Roughly one in seven inhabitants lives in a capital city, two in seven live in other urban areas, and four in seven in rural areas. A critical first step was finding robust methods to identify the urban poor in sample surveys. Wehrmeister et al. [10] showed that the bottom 40% of the population, according to household wealth distribution, can effectively characterize the poorest group. However, the heterogeneity of cities in terms of basic amenities among the poorest 40% is striking, with no clear subregional patterns emerging.

The paper by Amouzou et al. [11] provides compelling evidence of the erosion of the urban advantage in major cities compared to rural and other urban areas, driven primarily by faster progress in rural populations. This trend is particularly evident in eastern and southern Africa, where living in the city confers no neonatal or survival advantage compared to rural residents. In contrast, this advantage remains in parts of West and Central Africa.

However, the coverage of essential health interventions does not systematically align with these mortality trends. Blumenberg et al. [12] found that the gap between the poorest and richest populations in 38 cities gradually closed over time for delivery coverage, with the poorest 40% achieving over 80% coverage in 30 out of 38 cities in the most recent surveys. Despite this, the disparities in neonatal and under-five mortality and child stunting remain large. None of the 38 cities has already reached the SDG targets for under-five and neonatal mortality (25 and 12 per 1000 live births, respectively), and even the city non-poor fared poorly, with only three cities reaching the targets.

The special issue includes eight country-specific papers focusing on cities with populations ranging from 2.5 to 6.7 million: Accra [13], Bamako [14], Addis Ababa [15], Dakar [16], Dar es Salaam [17], Kampala [18], Lusaka [19], and Ouagadougou [20]. These papers examine various indicators of maternal and child health using multiple methods, including routine health information, health and demographic surveillance, and geospatial datasets. They confirm the utility of using survey analyses to document the disadvantage of the city poor compared to the non-poor, as well as the paradox of coexistence of high service coverage and high mortality, morbidity, and poor nutritional status among the urban poor.

For example, Bamako [14] and Lusaka [19] demonstrate how near-universal institutional delivery coverage among the poorest 40% (> 95%) can coexist with low caesarean section rates (below 5%), which indicates insufficient access to emergency care. WHO current guidelines suggest that caesarean rates below 10% are necessary to meet population needs. The paper on Dakar [16] goes a step further, showing a persistent gap in emergency care for the poorest women, with C-section rates of 4–5% compared to 8–9% for the wealthiest. The country’s free C-section policy in public services has been in place since 2005.

Several studies used routine health facility data to assess the coverage of interventions in districts that predominantly included slum settlements (Accra, Addis Ababa, Lusaka). These data complemented the survey findings but were affected by incomplete reporting and limitation in establishing appropriate denominators for coverage computations. Indicators of the quality of care, such as case fatality rates or emergency caesarean sections, provide further insights into the paradox of high contact coverage and high neonatal and child mortality rates, as shown in the Dar es Salaam study [17].

Few urban health and demographic surveillance systems exist in the region. The Ouagadougou paper [20] shows how child mortality inequalities have narrowed within the city due to malaria control and other infectious disease interventions. Bamako [14] and Ouagadougou [20] highlight the success of city strategies such as malaria control and free healthcare access for the poor.

As urbanization continues to reshape the region’s demographic landscape, capital cities must confront the challenges of rapid population growth, urban poverty, and inadequate access to essential health services. The findings point to significant issues with healthcare quality and living conditions affecting MNCH. An integrated approach to improving MNCH in cities must focus on the improvement of the quality of care, combined with addressing the social determinants of health inequities.

The results underscore the importance of targeted analyses to guide city programs toward the poorest populations, but they also highlight the limitations of current data, primarily drawn from national surveys. There is an urgent need for city-specific research on MNCH to guide policies and programs. Significant progress in urban areas, including cities, will contribute to accelerating progress toward SDG targets for maternal and child survival in sub-Saharan Africa.

Together, let us strive to build healthier, more equitable cities for all.
